# Quantum conductance of silicon-doped carbon wire nanojunctions

**DOI:** 10.1186/1556-276X-7-616

**Published:** 2012-11-07

**Authors:** Dominik Szczȩśniak, Antoine Khater, Zygmunt Ba̧k, Radosław Szczȩśniak, Michel Abou Ghantous

**Affiliations:** 1 Institute for Molecules and Materials UMR 6283, University of Maine, Ave. Olivier Messiaen, Le Mans, 72085, France; 2 Institute of Physics, Jan Długosz University in Czȩstochowa, Al. Armii Krajowej 13/15, Czȩstochowa, 42200, Poland; 3 Institute of Physics, Czȩstochowa University of Technology, Al. Armii Krajowej 19, Czȩstochowa, 42200, Poland; 4 Department of Physics, Texas A&M University, Education City, PO Box 23874, Doha, Qatar

**Keywords:** Nanoelectronics, Quantum wires, Electronic transport, Finite-difference methods, 85.35.-p, 73.63.Nm, 31.15.xf

## Abstract

Unknown quantum electronic conductance across nanojunctions made of silicon-doped carbon wires between carbon leads is investigated. This is done by an appropriate generalization of the phase field matching theory for the multi-scattering processes of electronic excitations at the nanojunction and the use of the tight-binding method. Our calculations of the electronic band structures for carbon, silicon, and diatomic silicon carbide are matched with the available corresponding density functional theory results to optimize the required tight-binding parameters. Silicon and carbon atoms are treated on the same footing by characterizing each with their corresponding orbitals. Several types of nanojunctions are analyzed to sample their behavior under different atomic configurations. We calculate for each nanojunction the individual contributions to the quantum conductance for the propagating *σ*, *Π*, and *σ*^∗^electron incidents from the carbon leads. The calculated results show a number of remarkable features, which include the influence of the ordered periodic configurations of silicon-carbon pairs and the suppression of quantum conductance due to minimum substitutional disorder and artificially organized symmetry on these nanojunctions. Our results also demonstrate that the phase field matching theory is an efficient tool to treat the quantum conductance of complex molecular nanojunctions.

## Background

Quantitative analysis of electronic quantum transport in nanostructures is essential for the development of nanoelectronic devices [1]. The monatomic *linear* carbon wire (MLCW) systems are expected in this context to have potentially interesting technological applications, in particular as connecting junction elements between larger device components [2]. In this respect, electronic quantum transport properties are the key features of such wire nanojunctions [3].

Carbon exists in nature under a wide range of allotropic forms as the two-dimensional graphene [4], the cage fullerenes [5], and the quasi one-dimensional carbon nanotubes [6]. These forms exhibit exceptional physical properties and can be considered as promising components for future nanodevices [7]. The discovery of MLCW, [8-14] turns the attention to another intriguing carbon allotropic form. In the experiment conducted recently by Jin et al. [14], MLCW was produced by directly removing carbon atoms row by row from the graphene sheets, leading to a relatively stable freestanding nanostructure.

At present, the available experimental data do not provide essential knowledge about the electronic properties of MLCW systems, and only theoretical studies shed some light on these properties. Furthermore, although the MLCW systems were investigated for a long time from the theoretical point of view [15-26], their interest was not highlighted until recently due to the open attention paid to other carbon allotropic forms. It has been shown in particular that from the structural point of view, MLCW can form either as cumulene wires (interatomic double bonds) or polyyne wires (alternating interatomic single and triple bonds) [14,17,19,27,28]. However, there is no straightforward answer as to which of these two structures is the favorable one; experimental studies do not give a satisfactory answer, and theoretical calculations yield provisions which depend on applied computational methods. Density functional theory (DFT) calculations predict double-bond structures [29,30], whereas *ab initio* Hartree-Fock (HF) results favor alternating bond systems [15-18,27]. This situation arises from the fact that DFT tends to underestimate bond alternation (second-order Jahn-Teller effect), while HF overestimates it [27].

More recently, first-principle calculations have indicated [31] that both structures are stable and present mechanical characteristics of a purely one-dimensional nanomaterial. Moreover, on the basis of the first-principle calculations [31-42], the cumulene MLCW wires are expected to be almost perfect conductors, even better than linear gold wires [29], while the corresponding polyyne wires are semiconducting [41]. It is also worth noting that the MLCW cumulene system may exhibit conductance oscillations with the even and odd numbers of the wire atoms [28,42].

**Figure 1:**
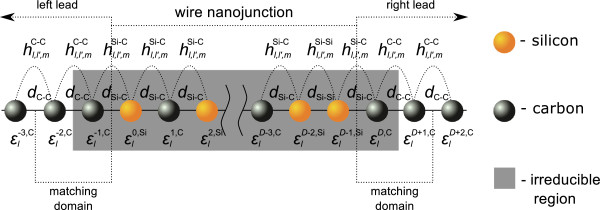
**Schematic representation of finite silicon-doped carbon wire nanojunction between two semi-infinite quasi one-dimensional carbon leads.** The irreducible region and matching domains are distinguished (please see subsection ‘Phase field matching theory’ in the ‘Methods’ section for more details). The binding energies for a given atomic site and the coupling terms between neighbor atoms with corresponding interatomic distances are depicted. The *n* and *n*^*′*^indices for the coupling parameters are dropped for simplicity.

In the present work, we consider in particular the problem of the electronic quantum transport across molecular nanojunctions made up of silicon-doped carbon wires, prepared in ordered or substitutionally disordered configurations as in the schematic representation of Figure 1, where the nanojunctions are between pure MLCW wire leads. This problem has not been considered previously and is still unsolved to our knowledge. The interest in the quantum transport of such nanojunctions arises from the fact that chemical defects or substitutional disorder may have a significant impact on their transport properties [43]. Chemical impurities doping the nanojunction may even allow the control of the transport for such nanostructures [44]. The properties of the nanoelectronic device and its functionality may hence be greatly affected or even built on such ordered and disordered configurations. The interest in silicon carbide, furthermore, stems from the fact that it is considered a good substrate material for the growth of graphene [45] and may produce interesting effects in its interactions with Si or C [46].

The electrons which contribute to transport present characteristic wavelengths comparable to the size of molecular nanojunctions, leading to quantum coherent effects. The transport properties of a given nanojunction are then described in terms of the Landauer-Büttiker theory [47,48], which relates transmission scattering to quantum conductance. Several approaches have been developed in order to calculate the scattering transmission and reflection cross sections in nanostructures, where the most popular are based on first-principle calculations [49,50] and semiempirical methods using the non-equilibrium Green’s function formalism [51,52].

In the present work, we investigate the electronic scattering processes on the basis of phase field matching theory (PFMT) [53,54], originally developed for the scattering of phonons and magnons in nanostructures [55-59]. Our theoretical method is based on appropriate phase matching of the Bloch states of ideal leads to the local states in the scattering region. In this approach, the electronic properties of the system are described in the framework of the tight-binding formalism (TB) which is widely exploited for electronic transport calculations [54,60-63] and for simulating the STM images of nanostructures [64,65]. In particular, we employ the appropriate Slater-Koster [66] type Hamiltonian parameters calculated on the basis of the Harrison’s tight-binding theory (HTBT) [67]. The PFMT method, which is formally equivalent to the method of non-equilibrium Green’s functions [68], can be considered consequently as a transparent and efficient mathematical tool for the calculation of the electronic quantum transport properties for a wide range of molecular-sized nanojunction systems.

The present paper is organized in the following manner. In the ‘Methods’ section, we give the detailed discussion of theoretical PFMT formalism. Our numerical results, which incorporate propagating and evanescent electronic states, are presented per individual lead modes in the ‘Results and discussion’ section. Also presented are the total conductance spectra; they are compared with results based on first-principle calculations when available. Finally, the discussion and conclusions are given in the ‘Conclusions’ section. Appropriate appendices which supplement the theoretical model are also presented.

## Methods

### Theoretical model and propagating states

The schematic representation of the system under study with an arbitrary nanojunction region is presented in Figure 1. With reference to the Landauer-Büttiker theory for the analysis of the electronic scattering processes [47,48], this system is divided into three main parts, namely the finite silicon-doped carbon wire nanojunction region, made up of a given composition of carbon (black) and silicon (orange) atoms, and two other regions to the right and left of the nanojunction which are semi-infinite quasi one-dimensional carbon leads. Moreover, for the purpose of quantum conductance calculations, the so-called irreducible region and the matching domains are depicted (see the ‘Phase field matching theory’ subsection for more details). Figure 1 is used throughout the ‘Methods’ section as a graphical reference for analytical discussion.

The system presented in Figure 1 is described by the general tight-binding Hamiltonian block matrix:

(1) $$H=\left[\begin{array}{ccccc} \ddots & \cdots & 0 & 0\\ \vdots & E_{N-1,N-1} & H_{N,N-1}^{†} & 0 & 0\\ 0 & H_{N,N-1} & E_{N,N} & H_{N+1,N}^{†} & 0\\ 0 & 0 & H_{N+1,N} & E_{N+1,N+1} & \vdots \\ & 0 & 0 & \cdots & \ddots \end{array}\right].$$

This is defined in general for a system of *N*_*x*_inequivalent atoms per unit cell, where *N*_*l*_denotes the number of basis orbitals per atomic site, assuming spin degeneracy. In Equation 1, ***E***_*i*,*j*_ denotes on-diagonal matrices composed of both diagonal $\varepsilon_{l}^{n,\alpha }$ and off-diagonal $h_{l,l^{\prime },m}^{n,n^{\prime },\beta }$ elements for a selected unit cell. In contrast, the ***H***_*i*,*j*_matrices contain only off-diagonal elements for interactions between different unit cells. The index *α*identifies the atom type, C or Si, on the *n*th site in a unit cell. Each diagonal element is characterized by the lower index *l* for the angular momentum state. The off-diagonal elements $h_{l,l^{\prime },m}^{n,n^{\prime },\beta }$ describe the *m*-type bond, (*m*=*σ*,*Π*), between *l* and *l*^*′*^ nearest-neighbor states. The index *β*identifies the types of interacting neighbors, C-C, Si-Si, or Si-C.

The $h_{l,l^{\prime },m}^{n,n^{\prime },\beta }$ elements are consistent with the Slater-Koster convention [66] and may be expressed in the framework of the HTBT [67] by the following:

(2) $$h_{l,l^{\prime },m}^{n,n^{\prime },\beta }=\eta_{l,l^{\prime },m}\frac{\hbar^{2}}{m_{e}d_{\beta }^{2}},$$

where $\eta_{l,l^{\prime },m}$ values are the dimensionless Harrison coefficients; *m*_*e*_, the electron mass in vacuum; and *d*_*β*_, the interatomic distance for interacting neighbors. Explicit forms of the ***E***_*i*,*j*_ and ***H***_*i*,*j*_ matrices are given in Appendix Appendix 1. The tight-binding parameter schemes are illustrated in Figure 1; however, it is noteworthy that the *n* and *n*^*′*^indices for coupling parameters are dropped for simplicity in this figure.

In our calculations, the single-particle electronic wave functions are expanded in the orthonormal basis of local atomic wave functions *ϕ*_*l*_(**r**) as follows:

(3) $$\Psi (r,k)=\underset{l,n,N}{\sum }c_{l}(r_{n}-R_{N},k)\phi_{l}(r-R_{N},k).$$

In Equation 3, ***k*** is the real wave vector; ***R***_*N*_, the position vector of the selected unit cell; and ***R***_*N*_, the position vector of the *n*th atom in the selected unit cell. For ideal leads, the wave function coefficients *c*_*l*_(***r***_*n*_−***R***_*N*_,***k***) are characterized under the Bloch-Floquet theorem in consecutive unit cells by the following phase relation:

(4) $$c_{l}(r_{n}-R_{N+1},k)=zc_{l}(r_{n}-R_{N},k),$$

where *z* is the phase factor

(5) $$z_{\pm }=e^{\pm ikR_{N}},$$

which corresponds here to waves propagating to the right (+) or to the left (−).

The electronic equations of motion for a leads unit cell, independent of *N*, may be expressed in a square matrix form, with an orthonormal minimal basis set of local wave functions as follows:

(6) $$(EI-M_{d})\times c(k,E)=0.$$

*E* stands for the electron eigenvalues, and ***I*** is the identity matrix, while the dynamical matrix ***M***_*d*_contains the Hamiltonian matrix elements and the *z* phase factors; ***c***(***k***,*E*) is the *N*_*x*_×*N*_*l*_ size vector defined as follows:

(7) $$c(k,E)=\left[\begin{array}{l} c_{s}(r_{1},k,E)\\ c_{p_{x}}(r_{1},k,E)\\ \vdots \\ c_{p_{y}}(r_{n},k,E)\\ c_{p_{z}}(r_{n},k,E) \end{array}\right]\equiv \left[\begin{array}{l} c_{l}(r_{1},k,E)\\ \vdots \\ c_{l}(r_{n},k,E) \end{array}\right].$$

Equation 6 gives the *N*_*x*_×*N*_*l*_eigenvalues with corresponding eigenvectors which determine the electronic structure of the lead system, where *l* under the vector ***c***_*l*_corresponds to *N*_*l*_=4 orbitals *s*,*p*_*x*_,*p*_*y*_,*p*_*z*_. Note that the choice of an orthonormal minimal basis set of local wavefunctions may result initially in an inadequate description of the considered electronic eigenvalues. However, as can be seen later, the proper choice of the TB on-site energies and coupling terms allows us to to obtain agreement with the DFT results. This is a systematic procedure in our calculations.

### Evanescent states

The complete description of electronic states on the ideal leads requires a full understanding of the propagating and evanescent electronic states on the leads. This arises because the silicon-doped nanojunction breaks the perfect periodicity of the infinite leads and forbids a formulation of the problem only in terms of the pure Bloch states as given in Equation 5. Depending on the complexity of a given electronic state, it follows that the evanescent waves may be defined by the phase factors for a purely imaginary wave vectors ***k***=*i****κ*** such that

(8) $$z=z_{\pm }=e^{\mp \kappa r_{n}},$$

or for complex wave vectors ***k***=***κ***_1_ + *i****κ***_2_such that

(9) $$z=z_{\pm }=e^{\mp (i\kappa_{1}-\kappa_{2})r_{n}}.$$

The phase factors of Equations 8 and 9 correspond to pairs of hermitian evanescent and divergent solutions on the leads. Only the evanescent states are physically considered where spatial evanescence occurs to the right and left, away from the nanojunction localized states. It is important to note that the *l*-type evanescent state corresponds to energies beyond the propagating band structure for this state.

The functional behavior of *z*(*E*) for the propagating and evanescent states on the leads may be obtained by various techniques. An elegant method presented previously for phonon and magnon excitations [59] is adapted here for the electrons. It is described on the basis of Equations 4 and 6 by the generalized eigenvalue problem for *z*:

(10) $$\begin{array}{lll} & \left[\left[\begin{array}{ll} EI-E_{N,N} & H_{N,N-1}\\ I & 0 \end{array}\right]-z\left[\begin{array}{ll} -H_{N,N-1}^{†} & 0\\ 0 & I \end{array}\right]\right]\; & \;\\ & \times \left[\begin{array}{l} c(R_{N},z,E)\\ c(R_{N-1},z,E) \end{array}\right]=0.\; \end{array}$$

Equation 10 gives the 2*N*_*x*_*N*_*l*_eigenvalues as an ensemble of *N*_*x*_*N*_*l*_pairs of *z* and *z*^−1^. Only solutions with |*z*|=1 (propagating waves) and |*z*|<1 (evanescent waves) are retained as physical ones. In Equation 10, ***k***is then replaced by the appropriate energy *E* variable. Furthermore, for systems with more than one atom per unit cell, the matrices ***H***_*N*,*N*−1_ and $H_{N,N-1}^{†}$ in this procedure are singular. In order to obtain the physical solutions, the eigenvalue problem of Equation 10 is reduced from the 2*N*_*x*_*N*_*l*_ size problem to the appropriate 2*N*_*l*_one, using the partitioning technique (please see Appendix Appendix 2).

### Phase field matching theory

The scattering problem at the nanojunction is considered next. An electron incident along the leads has a given energy *E* and wave vector ***k***, where *E*=*E*_*γ*_(***k***) denotes the available dispersion curves for *γ* = 1, 2,.., *γ* propagating eigenmodes, where *γ*corresponds to the total number of allowed solutions for the eigenvalue problem of phase factors in Equation 10. In any given energy interval, however, these may be evanescent or propagating eigenmodes and together constitute a complete set of available channels necessary for the scattering analysis.

The irreducible domain of atomic sites for the scattering problem includes the nanojunction domain itself, (*N*∈[0,*D*−1]), and the atomic sites on the left and right leads which interact with the nanojunction, as in Figure 1. This constitutes a necessary and sufficient region for our considerations, i.e., any supplementary atoms from the leads included in the calculations do not change the final results. The scattering at the boundary yields then the coherent reflected and transmitted fields, and in order to calculate these, we establish the *system* of equations of motion for the atomic sites (*N*∈[−1,*D*]) of the irreducible nanojunction domain.

This procedure leads to the following general matrix equation:

(11) $$M_{\mathrm{nano}}\times V=0.$$

***M***_*nano*_ is a (*D* + 2)×(*D* + 4) matrix composed of the block matrices $\left(EI-E_{N,N}-H_{N,N-1}-H_{N,N-1}^{†}\right)$, and the state vector ***V***of dimension *D* + 4 is given as follows:

(12) $$V=\left[\begin{array}{l} c_{l}(r_{1}-R_{-2},E)\\ \vdots \\ c_{l}(r_{n}-R_{-2},E)\\ \vdots \\ \vdots \\ c_{l}(r_{1}-R_{D+1},E)\\ \vdots \\ c_{l}(r_{n}-R_{D+1},E) \end{array}\right].$$

Since the number of unknown coefficients in Equation 11 is always greater than the number of equations, such a set of equations cannot be solved directly.

Assuming that the incoming electron wave propagates from left to right in the eigenmode *γ* over the interval of energies *E*=*E*_*γ*_, the field coefficients on the left and right sides of the irreducible nanojunction domain may be written as follows:

(13) $$\begin{array}{lll} c_{l}^{L}(r_{n}-R_{N},z_{\gamma },E_{\gamma }) & =c_{l}(r_{n},z_{\gamma },E_{\gamma })z_{\gamma }^{-N}\; & \;\\ & \;\;\;+\overset{\Gamma }{\underset{\gamma^{\prime }}{\sum }}c_{l}(r_{n},z_{\gamma^{\prime }},E_{\gamma })z_{\gamma^{\prime }}^{N}r_{\gamma ,\gamma^{\prime }}(E_{\gamma })\;\;\; & \;\\ & \;\;\;\text{for}\;\;N\leq -1,\; \end{array}$$

(14) $$\begin{array}{lll} c_{l}^{R}(r_{n}-R_{N},z_{\gamma },E_{\gamma }) & =\overset{\Gamma }{\underset{\gamma^{\prime }}{\sum }}c_{l}(r_{n},z_{\gamma^{\prime }},E_{\gamma })z_{\gamma^{\prime }}^{N}t_{\gamma ,\gamma^{\prime }}(E_{\gamma })\;\;\; & \;\\ & \;\;\;\text{for}\;\;N\geq D,\; \end{array}$$

where *γ*^*′*^∈*Γ*is an arbitrary channel into which the incident electron wave scatters, and ***c***_*l*_(***r***_*n*_,*z*_*γ*_,*E*_*γ*_) denotes the the eigenvector of the lead dynamical matrix of Equation 6 for the inequivalent site *n* at *z*_*γ*_ and *E*_*γ*_. The terms $r_{\gamma ,\gamma^{\prime }}$ and $t_{\gamma ,\gamma^{\prime }}$ denote the scattering amplitudes for backscattering and transmission, respectively, from the *γ* into the *γ*^*′*^eigenmodes and constitute the basis of the Hilbert space which describes the reflection and transmission processes.

Equations 13 and 14 are next used to transform the (*D* + 2)×(*D* + 4) matrix of the system of equations of motion, Equation 11, into an inhomogeneous (*D* + 2)×(*D* + 2) matrix for the scattering problem. This procedure leads to the new form of the following vector:

(15) $$\begin{array}{lll} V & =\left[\begin{array}{lllllll} z^{2} & 0 & \cdots & \cdots & \cdots & \cdots & 0\\ z & 0 & \vdots \\ 0 & 1 & \vdots \\ \vdots & \ddots & \vdots \\ \vdots & 1 & \vdots \\ \vdots & \ddots & \vdots \\ \vdots & 1 & 0\\ \vdots & 0 & z\\ 0 & \cdots & \cdots & \cdots & \cdots & 0 & z^{2} \end{array}\right]\; & \;\\ & \;\;\;\times \left[\begin{array}{l} r_{\gamma ,\gamma^{\prime }}\\ c_{l}(r_{1}-R_{0},E_{\gamma })\\ \vdots \\ c_{l}(r_{n}-R_{0},E_{\gamma })\\ \vdots \\ \vdots \\ c_{l}(r_{1}-R_{D-1},E_{\gamma })\\ \vdots \\ c_{l}(r_{n}-R_{D-1},E_{\gamma })\\ t_{\gamma ,\gamma^{\prime }} \end{array}\right]+\left[\begin{array}{l} c_{l}(r_{1},z_{\gamma },E_{\gamma })z_{\gamma }^{-2}\\ \vdots \\ c_{l}(r_{n},z_{\gamma },E_{\gamma })z_{\gamma }^{-2}\\ c_{l}(r_{1},z_{\gamma },E_{\gamma })z_{\gamma }^{-1}\\ \vdots \\ c_{l}(r_{n},z_{\gamma },E_{\gamma })z_{\gamma }^{-1}\\ 0\\ \vdots \\ \vdots \\ 0 \end{array}\right].\; \end{array}$$

The rectangular sparse matrix in Equation 15 has the (*D* + 4)×(*D* + 2) size. The vectors $r_{\gamma ,\gamma^{\prime }}$ and $t_{\gamma ,\gamma^{\prime }}$ are column vectors of the backscattering and transmission Hilbert basis.

Substituting Equation 15 into Equation 11 yields an inhomogeneous system of equations as follows:

(16) $$M\times \left[\begin{array}{l} r_{\gamma ,\gamma^{\prime }}\\ c_{l}(r_{1}-R_{0},E_{\gamma })\\ \vdots \\ c_{l}(r_{n}-R_{0},E_{\gamma })\\ \vdots \\ \vdots \\ c_{l}(r_{1}-R_{D-1},E_{\gamma })\\ \vdots \\ c_{l}(r_{n}-R_{D-1},E_{\gamma })\\ t_{\gamma ,\gamma^{\prime }} \end{array}\right]=-\left[\begin{array}{l} M_{1}^{\mathrm{in}}\\ M_{2}^{\mathrm{in}}\\ 0\\ \vdots \\ 0 \end{array}\right].$$

In Equation 16, ***M*** is the *matched*(*D* + 2)×(*D* + 2) square matrix, and the vector of dimension (*D* + 2) which incorporates the $M_{1}^{\mathrm{in}}$ and $M_{2}^{\mathrm{in}}$ elements, regroups the inhomogeneous terms of the incident wave. The explicit forms of the ***M*** matrix elements and and $M_{N}^{\mathrm{in}}$ vectors are presented in Appendix Appendix 3.

In practice, Equation 16 can be solved using standard numerical procedures, over the entire range of available electronic energies, yielding the coefficient ***c***_*l*_ for atomic sites on the nanojunction domain itself as well as the *γ*reflection $r_{\gamma ,\gamma^{\prime }}(E)$ and the *γ*transmission $t_{\gamma ,\gamma^{\prime }}(E)$ coefficients.

The reflection and transmission coefficients give the reflection $r_{\gamma ,\gamma^{\prime }}(E)$ and transmission $t_{\gamma ,\gamma^{\prime }}(E)$ probabilities, respectively, by normalizing with respect to their group velocities *v*_*γ*_ in order to obtain the unitarity of the scattering matrix as follows:

(17) $$R_{\gamma ,\gamma^{\prime }}(E)=\frac{v_{\gamma^{\prime }}}{v_{\gamma }}{\left|r_{\gamma ,\gamma^{\prime }}(E)\right|}^{2},$$

(18) $$T_{\gamma ,\gamma^{\prime }}(E)=\frac{v_{\gamma^{\prime }}}{v_{\gamma }}{\left|t_{\gamma ,\gamma^{\prime }}(E)\right|}^{2},$$

where *v*_*γ*_≡*v*_*γ*_(*E*) denotes the group velocity of the incident electron wave in the eigenmode *γ*. The group velocities are calculated by a straightforward procedure as in Appendix Appendix 4. For evanescent eigenmodes, $v_{\gamma^{\prime }}=0$. Although the evanescent eigenmodes do not contribute to the electronic transport, they are required for the complete description of the scattering processes.

Furthermore, using Equations 17 and 18, the overall reflection probability, *R*_*γ*_(*E*), for an electron incident in the *γ* eigenmode and the total electronic reflection probability, *R*(*E*), from all the eigenmodes may be expressed, respectively, as follows:

(19) $$R_{\gamma }(E)=\overset{\Gamma }{\underset{\gamma^{\prime }}{\sum }}R_{\gamma ,\gamma^{\prime }}(E)\;\;\text{and}\;\;R(E)=\overset{\Gamma }{\underset{\gamma }{\sum }}R_{\gamma }(E).$$

Similarly, for transmission probabilities, we may write the equivalent equations as follows:

(20) $$T_{\gamma }(E)=\overset{\Gamma }{\underset{\gamma^{\prime }}{\sum }}T_{\gamma ,\gamma^{\prime }}(E)\;\;\text{and}\;\;T(E)=\overset{\Gamma }{\underset{\gamma }{\sum }}T_{\gamma }(E).$$

The *T*_*γ*_(*E*) and *T*(*E*) probabilities are very important for the electronic scattering processes since they correspond directly to the experimentally measurable observables. Likewise, the total transmission *T*(*E*_*γ*_) allows to calculate the overall electronic conductance. In this work, we assume the zero-bias limit and write the total conductance in the following way:

(21) $$G(E_{F})=G_{0}T(E_{F}).$$

In Equation 21, *G*_0_ is the conductance quantum and equals 2*e*^2^/h. Due to the Fermi-Dirac distribution, *G*(*E*_*F*_) is calculated at the Fermi level of the perfect lead band structure since electrons only at this level give the important contribution to the electronic conductance. The Fermi energy can be determined using various methods where, in the present work, *E*_*F*_ is calculated as the basis of the density of state calculations.

## Results and discussion

### The tight-binding model and basic electronic properties

In this section, we present the results of our model calculations for the electronic structure of carbon, silicon, and silicon carbide wires under study. Our results are validated by comparison with DFT calculations [29,69], which allow us to establish unambiguously our choice of the tight-binding parameters for these systems.

In principle, we can develop our model calculations for the nanojunctions and their leads using any adequate type of orbitals; even a single orbital suffices to calculate the electronic quantum transport for carbon nanojunctions [44]. However, this approximation is inadequate for silicon atoms. To treat both types of atoms on the same footing, we thus characterize the atoms by the electronic states 2*s* and 2*p* for carbon and by 3*s* and 3*p* for silicon. Such a scheme gives us four different orbitals, namely *s*, *p*_*x*_, *p*_*y*_, and *p*_*z*_, for both types of atoms.

**Figure 2:**
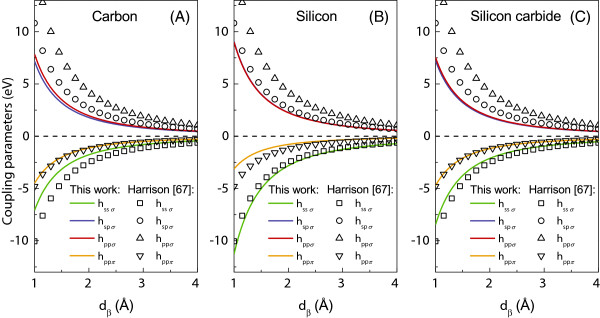
**The nearest-neighbor tight-binding coupling parameters with the interatomic distance (A, B, C).** The curves represent our calculated TB results in comparison with those calculated using the Harrison parameters (squares, triangles, circles).

In the present work, our TB parameters are effectively rescaled from the Harrison’s data in order to match our model calculations for the electronic structure with those given by the DFT. The utilized TB parameters are presented in Table 1 in comparison with the values given by Harrison. It is worthy to note that the values of the on-site Hamiltonian matrix elements $\varepsilon_{p}^{n,\alpha }$ are identical for states *p*_*x*_, *p*_*y*_, and *p*_*z*_. The off-diagonal distance-dependent $h_{l,l^{\prime },m}^{n,n^{\prime },\beta }$ elements are calculated on the basis of Equation 2. For symmetry considerations, these latter elements are positive or negative, also *h*_*s*,*p*,*σ*_ = *η*_*s*,*p*,*σ*_ = 0 and *h*_*p*,*p*,*σ*_ = *η*_*p*,*p*,*σ*_ = 0, for *p*_*y*_ and *p*_*z*_, and $h_{p,p,\Pi }=h_{p_{y},p_{y},\Pi }=h_{p_{z},p_{z},\Pi }=h_{p_{x},p_{x},\Pi }=0$[70]. Table 1 is supplemented for the reader by Figure 2 which gives the dependence of the hopping integrals with distance as calculated in the present paper (continuous curves), in comparison with the Harrison’s data (open symbols). 

**Table 1:** Tight-binding parameters and Harrison’s dimensionless coefficients proposed in this work and compared with original values

	**Harrison TB parameters**			**Present TB parameters**		
*α*	C	Si		C	Si	
*ε*_*s*_	−19.38	−14.79		−18.89	−13.5	
*ε*_*p*_	−11.07	−7.59		−10.94	−8.38	
*β*	C-C	Si-Si	Si-C	C-C	Si-Si	Si-C
*η*_*s*,*s*,*σ*_	−1.32	−1.32	−1.32	−0.93	−1.48	−1.11
*η*_*s*,*p*,*σ*_	1.42	1.42	1.42	0.94	1.19	0.95
*η*_*p*,*p*,*σ*_	2.22	2.22	2.22	1.03	1.18	0.99
*η*_*p*,*p*,*Π*_	−0.63	−0.63	−0.63	−0.59	−0.41	−0.62
*β*	C-C	Si-Si	Si-C	C-C	Si-Si	Si-C
*h*_*s*,*s*,*σ*_	−5.95	−2.08	−3.70	−4.19	−2.33	−3.11
*h*_*s*,*p*,*σ*_	6.40	2.24	3.98	4.23	1.87	2.66
*h*_*p*,*p*,*σ*_	10.01	3.50	6.22	4.64	1.86	2.77
*h*_*p*,*p*,*Π*_	−2.84	−0.99	−1.77	−2.66	−0.65	−1.74
*β*	C-C	Si-Si	Si-C	C-C	Si-Si	Si-C
*d*_*β*_	1.3	2.2	1.649	1.3	2.2	1.649

The values of the tight-binding parameters, $\varepsilon_{l}^{n,\alpha }$ and $h_{{l,l}^{\prime },m}^{{n,n}^{\prime },\beta }$ (in eV), and Harrison’s dimensionless coefficients, $\eta_{l,l^{\prime },m}$, as proposed in this work and compared with the original values by Harrison [67]. Please note that the distance-dependent $h_{{l,l}^{\prime },\sigma }^{{n,n}^{\prime },\beta }$ parameters are computed for the appropriate interatomic spacings ***d***_***β***_(in Å), tabulated below, and assumed after the works of Tongay et al. [29] and Bekaroglu et al. [69]. In order to keep table transparent indices, ***n***and ***n***^*′*^ for $\varepsilon_{l}^{n,\alpha }$ and $h_{{l,l}^{\prime },m}^{{n,n}^{\prime },\beta }$ are omitted.

Figure 2 clearly indicates the fact that qualitatively, both Harrison’s and our rescaled coupling parameters for silicon, carbon and diatomic silicon carbide wires, present the same functional behavior, confirming the desired conservation of their physical character. However, most of the rescaled coupling parameters have somehow smaller values than those initially proposed by Harrison; this trend can be also traced in Table 1 for the onsite parameters. This difference stems from the influence of the low-coordinated systems are considered here, whereas the initial Harrison values are given to match tetrahedral phases [67]. Another general observation can be made for the tight-binding parameters of the *σ*-type interactions (the *h*_*s*,*p*,*σ*_and *h*_*p*,*p*,*σ*_ones), which present much closer values over the considered interatomic distance range than in the case of Harrison’s data.

**Figure 3:**
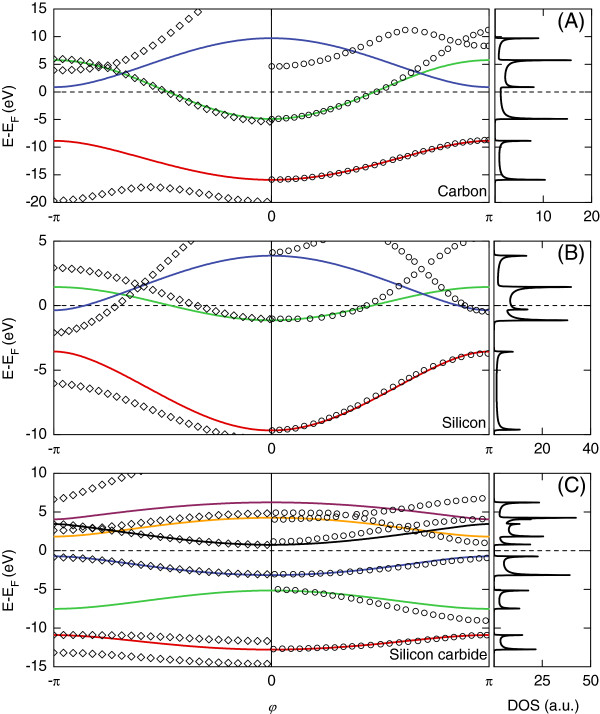
**Electronic structures of carbon (A), silicon (B), and diatomic silicon carbide (C).** These structures are for infinite linear atomic wires presented over the first Brillouin zone *φ*=*kd*∈[−*ΠΠ*. Our calculated results (continuous curves), represented by a color scheme (details in the text), are compared on the right-hand side with the first-principle results (closed circles, *φ*∈[0,*Π*) [29,69] and on the left-hand side with results calculated using Harrison TB parameters [67] (diamonds, *φ*∈[−*Π*,0]). Our calculated Fermi levels are given as the zero-reference energies, and the calculated electronic DOS in arbitrary units are presented in the right-hand column.

Our calculated electronic band structures for silicon, carbon, and diatomic silicon carbide infinite wires (continuous curves) are presented in Figure 3 in comparison with the DFT results [29,69] as in the right-hand side of the figures. We note for the carbon and silicon structures that our TB parameters correctly reproduce the DFT results up to energies slightly above the Fermi level. Electronic branches in the regions of high energies are in qualitative agreement. In the case of the diatomic silicon carbide structure, some of the electronic states perfectly match the DFT results even for high-energy domains. The left-hand side of Figure 3 compares our results (continuous curves) with those from the older TB values given by Harrison (open symbols); as is seen, our TB parameters constitute the most optimal set for the electronic transport calculations since their corresponding electronic band structures conform to the appropriate energy ranges highlighted by the DFT results and, what is even more important, correctly reproduce the Fermi level.

In Figure 3A,B for silicon and carbon, the red and blue colors correspond, respectively, to the *σ* and *σ*^∗^ bands. These arise from the *sp*_*x*_orbital hybrids where the lowest lying bands are always occupied by two electrons. Bands marked by the red color have the *Π* character and are degenerate. Their origin in the *p*_*y*_and *p*_*z*_orbitals allows them to hold up to four electrons. In Figure 3C for the diatomic silicon carbide, starting from the band structure minimum, consecutive bands have their origin in the following orbitals: carbon 3*s* (red band), silicon 3*s* (green band), carbon 3*p* (blue and black bands), and silicon 3*p* (orange and violet bands). The blue and orange colors for the silicon carbide electronic structure indicate two doubly degenerate *Π*-type bands.

The metallic or insulating character of the considered atomic wires, following the Fermi level, is appropriate only when the wires are infinite. It is well known that this character can change for the case of finite size wires with a limited number of atoms or due to the type and quality of the leads.

### Numerical characteristics for the carbon leads

In general, the infinite carbon wires which are considered as the leads in our work, present electronic band structure characteristics which incorporate not only propagating (see Figure 3A), but also evanescent states. Both of these types of states, which are derivable from the generalized eigenvalue problem as presented in Equation 10, constitute a complete set over the allowed energies for the electrons incident along the leads, which can be further scattered at the considered nanojunction. This complete set of eigenstates is used as the basis for the numerical calculations of the quantum conductance presented in the ‘Transport properties’ subsection.

**Figure 4:**
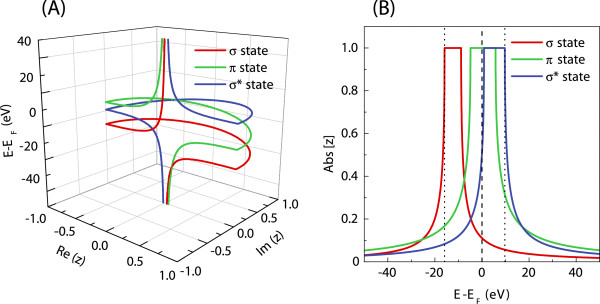
**Three-dimensional representation of the functionals *z(E) *and the evolution of their absolute values for carbon leads. **(**A**) Three-dimensional representation of the functionals *z*(*E*) on a complex plane and (**B**) the evolution of their absolute values as a function of energy for carbon leads. The color scheme here is the same as that for carbon in Figure 3A.

Figure 4A presents the three-dimensional representation of the solutions of Equation 10 as a set of generalized functionals *z*(*E*) for the *σ*, *σ*^∗^, and *Π*electronic states of the carbon leads. As described by Equations 5, 8, and 9, the eigenstates in Figure 4A characterized by |*z*|=1 correspond to the propagating electronic waves described by the real wave vectors, whereas those by |*z*|<1 correspond to the evanescent and divergent eigenstates for the complex wave vectors. Furthermore, for convenience, the corresponding moduli of the complex *z* factors are presented in Figure 4B. Note that |*z*|=1 solutions may be grouped into pairs for the two directions of propagation linked by time-reversal symmetry. Due to the fact that each of these two solutions provides the same information, we consider waves propagating only from left to right. However, this is not true for the |*z*|<1 solutions which are always considered for both left and right as spatially evanescent. As can be seen in Figure 4, the generalized results for *σ*, *σ*^∗^, and *Π* states are represented by the same colors as the corresponding states in Figure 3A, following their propagating character for |*z*|=1, and further extended to the physically |*z*|<1 evanescent solutions.

Figure 4 provides a more complete description for the electronic states of a given system compared to a typical band structure representation as in Figure 3, since both the propagating and evanescent states are shown. Such a general representation clearly indicates the importance of the evanescent eigenstates for a full description of the scattering problem presented in the ‘Transport properties’ subsection. The energies considered in our calculations correspond to the range within the band structure boundaries, marked by two vertical dotted lines in Figure 4B. As a consequence, not only the propagating states, but also the evanescent solutions are included in the quantum conductance calculations in the ‘Transport properties’ subsection.

### Transport properties

**Figure 5:**
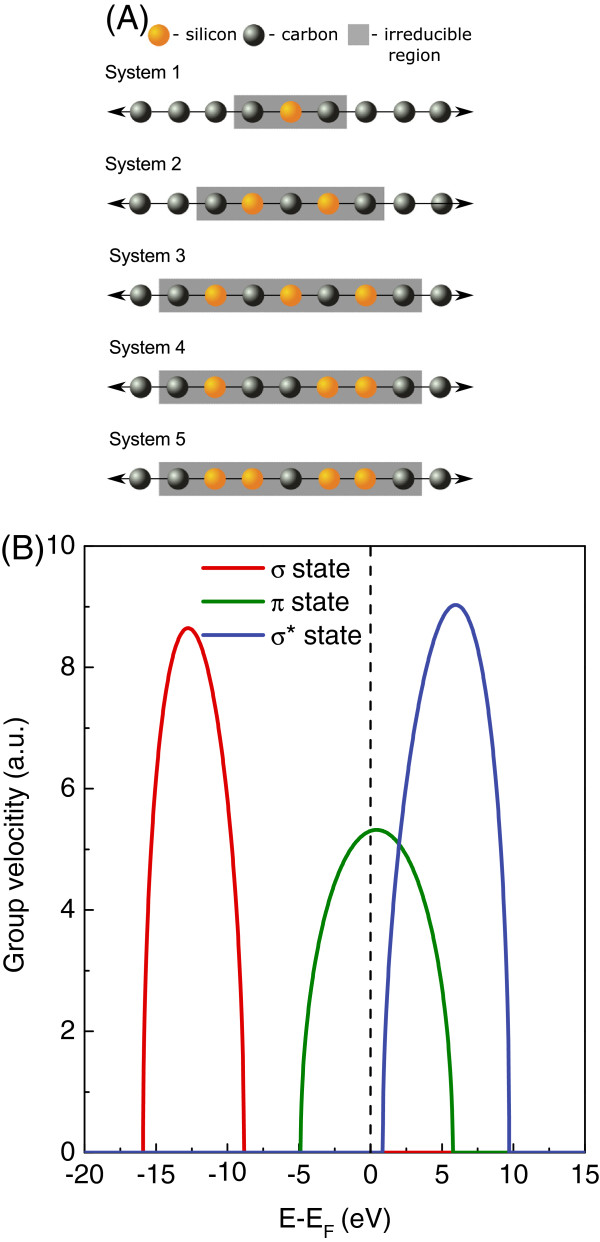
**Schematic representation of the five nanojunction systems and group velocities for propagating band structure modes. (A)** Schematic representation of the five nanojunction systems composed of silicon and carbon atoms between one-dimensional carbon leads considered in the present work. The irreducible domains are marked by the shaded grey areas, whereas for the other cases, only the irreducible domains are shown. **(B)** The group velocities for the propagating band structure modes on the carbon leads.

In this subsection, the electronic transport properties of nanojunction systems composed of silicon-doped carbon wires between carbon leads are calculated using the PFMT method. Figure 5A presents a number of these systems where we indicate the irreducible domains by the shaded grey areas. Note that these systems are always composed of finite nanojunction regions of silicon and carbon atoms, coupled with two carbon semi-infinite leads. The first three systems of Figure 5 correspond to periodic diatomic silicon carbide nanojunctions composed of 1, 2, and 3 Si-C atomic pairs, respectively. The next system corresponds to a nanojunction with a substitutional disorder, composed of three carbon and three silicon atoms. The last is a symmetric nanojunction of five silicon atoms and only one carbon atom in the middle. Figure 5B presents the group velocities of electrons in the carbon leads.

**Figure 6:**
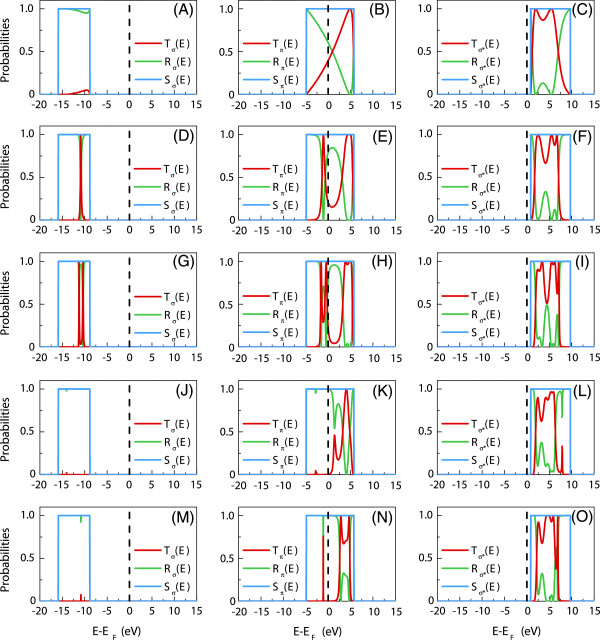
**Transmission and reflection probabilities across five types of silicon-doped carbon wires between two semi-infinite one-dimensional carbon leads.** The arrangement of the figure is as follows: (**A, B, C**) for case 1, (**D, E, F**) for case 2, (**G, H, I**) for case 3, (**J, K, L**) for case 4, and (**M, N, O** for case 5. The Fermi level is set at the zero-energy reference position.

The calculated transmission and reflection scattering cross sections for each of the four available transport channels are presented in Figure 6. Each row of the figure corresponds to a nanojunction system (NS) as follows: Figure 6A,B,C for NS 1, Figure 6D,E,F for NS 2, Figure 6G,H,I for NS 3, Figure 6J,K,L for NS 4, and Figure 6M,N,O for NS 5. The red and green continuous curves represent the transmission and reflection spectra, respectively. The blue histograms correspond to the free electronic transport on the carbon leads, i.e., to the electronic transport on the perfect infinite quasi one-dimensional carbon wire over the different propagating states. These histograms constitute the reference to the unitarity condition which is used systematically as a check on the numerical results. The leads’ Fermi level is marked by a dashed line and set as a zero-energy reference. Under the zero-bias limit, the total conductance is calculated at this Fermi level.

In Figure 6, the transmission spectra present strong scattering resonances, showing an increasing complexity with the increasing size and configurational order of the nanojunctions. The valence *σ*state exhibits negligible transmission for all of the considered nanojunctions. The degenerate *Π* states and the *σ*^∗^state present in contrast the finite transmission spectra. However, it is only the *Π* states which cross the Fermi level, giving rise to electronic conductance across the nanojunction within the zero-bias limit.

In particular, the first three considered systems represent increasing lengths of the diatomic silicon carbide nanojunction with the increasing number of ordered Si-C atomic pairs. The transmission at the Fermi level for these systems is nonzero (see Figure 3C), which contrasts with the insulating character of the infinite silicon carbide wire. One can connect this finite transmission to the indirect bandgap (*Δ*) around the Fermi level for the diatomic silicon-carbide infinite wire (for more details, please see Figure 3C). This gap, *Δ*∼1*.*5 eV, is indeed related to the difference between the binding energies of the silicon and carbon atoms and corresponds to an effective potential barrier for the propagating *Π*-state electrons. As the wire length increases by adding Si-C atomic pairs, as for systems 1 to 3 of Figure 5B, the transmission decreases due to cumulative barrier effects. We note that a similar effect for the monovalent diatomic copper-cobalt wire nanojunctions has been observed in a previous work [54].

Furthermore, it is instructive to compare the scattering spectra for the degenerate *Π*states, for nanojunction systems 3 and 4. These two systems contain identical numbers of silicon and carbon atoms; however, system 3 is an ordered configuration of Si-C pairs, whereas system 4 presents substitutional disorder of the atoms. It is seen that the disorder suppresses the conductance of the *Π*-state electrons at the Fermi level within the zero-bias limit. Another general observation can be made from the results for nanojunction system 5 which contains more silicon than carbon atoms. Despite the finite size of this system, which is comparable to system 4, and despite the structural symmetry of its atomic configuration, the electronic transmission is suppressed at the Fermi level within the zero-bias limit. This implies that one of the main observations of our paper is that structural symmetry on the nanojunction is not a guarantee for finite transmission in the case of the multivalence diatomic wire nanojunctions.

Figure 6 also shows that the transmission spectra for the *σ*^∗^ state are close to unity over a significant range of energies from approximately 1 to 7 eV for all of the five nanojunction systems. This result may prove useful for the electronic conductance across silicon-doped carbon nanojunctions under finite bias voltages.

**Figure 7:**
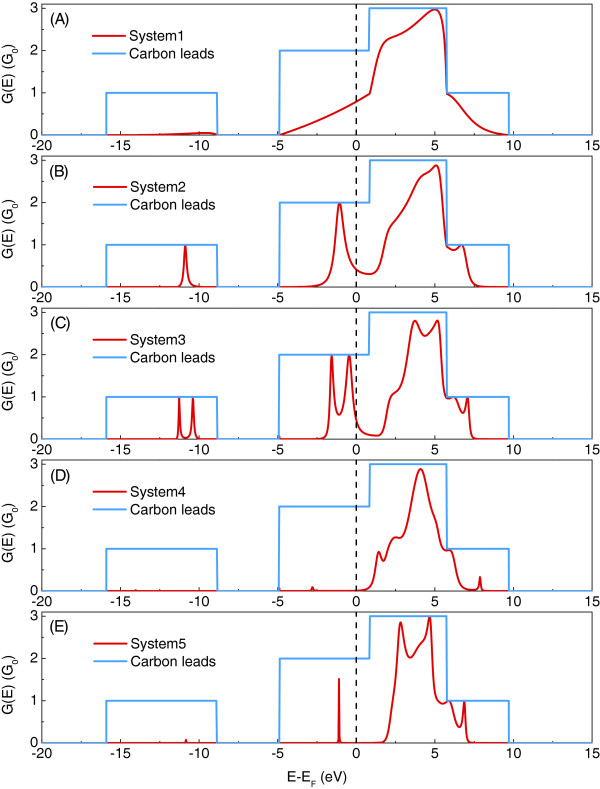
**Total electronic conductance.** Total electronic conductance *G*(*E*) (**A, B, C, D, E**) as a function of energy *E* in units of *G*_0_=2*e*^2^/h for silicon-doped carbon wires. See text for details.

In Figure 7, we present the total electronic conductance *G*(*E*) as a function of energy *E* and in units of *G*_0_=2*e*^2^/h for the considered nanojunction systems of a given length as depicted in Figure 5 (red). Moreover, the perfect electronic conductance on the carbon leads (blue) is given in comparison and constitutes effectively the conductance of the infinite and perfect quasi one-dimensional carbon wire. In Figure 7, the Fermi level is indicated by the dashed line as a zero-reference energy, and *G*(*E*) is calculated from all the contributing eigenstates of Figure 6, including the two degenerate *Π* states.

We note that the conclusions given for the results presented in Figure 6 are also followed by the more general representation of the electronic transport depicted in Figure 7. Furthermore, the results presented in Figure 7 confirm that only the electrons incident from the leads in the *Π* states are responsible for the electronic conductance at the zero-bias limit, which is readable from the Fermi level position. However, for all considered systems, the conductance at the Fermi level is theoretically limited to the value of 2 *G*_0_, and the biggest conductance maxima close to the perfect infinite carbon wire value of 3 *G*_0_can be observed only in the energy interval from approximately 1 to 7 eV hence for energies above the Fermi level. Once again, this follows our previous observations for the transmission results for the *Π*states concluded from Figure 6. Nonetheless, only on the basis of the results presented in Figure 7 can we note that due to the summation over all possible state contributions which constitute the *G*(*E*) spectra, not only the *σ*^∗^-state electrons, but also some of those in the degenerate *Π*states contribute to the high conductance values in the cited energy intervals. This important observation proves that the *σ*^∗^- and *Π*-state electrons are of crucial importance for both the zero-bias quantum conductance of the silicon-doped carbon wires and the possible finite bias ones. This implies that the use of only a single orbital for the description of the carbon atoms will result in an inadequate description of the transport processes across low-coordinated systems containing these atoms.

## Conclusions

In the present work, the unknown properties of the quantum electronic conductance for nanojunctions made of silicon-doped carbon wires between carbon leads are studied in depth. This is done using the phase field matching theory and the tight-binding method. The local basis for the electronic wave functions is assumed to be composed of four different atomic orbitals for silicon and carbon, namely the *s*, *p*_*x*_, *p*_*y*_, and *p*_*z*_states.

In the first step, we calculate the electronic band structures for three nanomaterials, namely the one-dimensional infinite wires of silicon, carbon, and diatomic silicon carbide. This permits a matching comparison with the available corresponding DFT results, with the objective to select the optimal TB parameters for the three nanomaterials.

This optimal set of the tight-binding parameters is then used to calculate the electronic conductance across the silicon-doped carbon wire nanojunctions. Five different nanojunction cases are analyzed to sample their behavior under different atomic configurations. We show that despite the nonconducting character of the infinite silicon carbide wires, its finite implementation as nanojunctions exhibit a finite conductance. This outcome is explained by the energy difference between the binding energies of the silicon and carbon atoms, which correspond to an effective potential barrier for the degenerate *Π*-state electrons transmitted across the nanojunction under zero-bias field.

The conductance effects that may arise due to minimal substitutional disorder and to artificially organize symmetry considerations on the silicon carbide wire nanojunction are also investigated. By exchanging the positions of two silicon and carbon atoms on an initial nanojunction to generate a substitutional disorder, we show that the total quantum conductance is suppressed at the Fermi level. This is in sharp contrast with the finite and significant conductance for the initial atomically ordered nanojunction with periodic configurations of the silicon and carbon atoms. Also, the analysis of a silicon carbide nanojunction of a comparable size as the one above, presenting symmetry properties, shows that quantum conductance is suppressed at the Fermi level.

In summary, we note that the biggest maxima of the conductance spectra for the zero-bias limit can be observed for high energies for all of the considered systems. This conclusion reveals the fact that electrons incident from the leads in both *σ*^∗^and *Π*states are crucial for the considerations of the electronic transport properties of the silicon-doped carbon wire nanojunctions.

## Appendix 1

### Explicit forms of the ***E***^***i***,*j*^and ***H***^***i***,*j*^matrices

The explicit forms of the submatrices of Equation 1 are given in the following manner:

(22) $$E_{i,j}=\left[\begin{array}{lllll} \varepsilon_{1} & h_{2,1}^{†} & \cdots & \cdots & h_{n,1}^{†}\\ h_{2,1} & \varepsilon_{2} & \ddots & & \vdots \\ \vdots & \ddots & \ddots & \ddots & \vdots \\ \vdots & & \ddots & \varepsilon_{n-1} & h_{n,n-1}^{†}\\ h_{n,1} & \cdots & \cdots & h_{n,n-1} & \varepsilon_{n} \end{array}\right],$$

and

(23) $$H_{i,j}=\left[\begin{array}{llllll} 0 & \cdots & h_{1,2} & \cdots & h_{1,n-1} & h_{1,n}\\ \vdots & \ddots & \ddots & \ddots & h_{2,n-1} & h_{2,n}\\ 0 & \ddots & \ddots & \ddots & \ddots & \vdots \\ \vdots & \ddots & \ddots & \ddots & \ddots & h_{n,n}\\ 0 & 0 & \ddots & \ddots & \ddots & \vdots \\ 0 & 0 & \cdots & 0 & \cdots & 0 \end{array}\right],$$

where

(24) $$\varepsilon_{i^{\prime },j^{\prime }}=\left[\begin{array}{lllll} \varepsilon_{s}^{n,\alpha } & 0 & \cdots & \cdots & 0\\ 0 & \varepsilon_{p_{x}}^{n,\alpha } & \ddots & & \vdots \\ \vdots & \ddots & \ddots & \ddots & \vdots \\ \vdots & & \ddots & \varepsilon_{l-1}^{n,\alpha } & 0\\ 0 & \cdots & \cdots & 0 & \varepsilon_{l}^{n,\alpha } \end{array}\right],$$

and

(25) $$h_{i^{\prime },j^{\prime }}=\left[\begin{array}{lllll} h_{s,s,\sigma }^{n,n^{\prime },\beta } & h_{s,p_{x},\sigma }^{n,n^{\prime },\beta } & \cdots & \cdots & h_{s,l^{\prime },m}^{n,n^{\prime },\beta }\\ h_{p_{x},s,\sigma }^{n,n^{\prime },\beta } & h_{p_{x},p_{x},\sigma }^{n,n^{\prime },\beta } & \ddots & & \vdots \\ \vdots & \ddots & \ddots & \ddots & \vdots \\ \vdots & & \ddots & h_{l-1,l^{\prime }-1,m}^{n,n^{\prime },\beta } & h_{l-1,l^{\prime },m}^{n,n^{\prime },\beta }\\ h_{l,s,m}^{n,n^{\prime },\beta } & \cdots & \cdots & h_{l,l^{\prime }-1,m}^{n,n^{\prime },\beta } & h_{l,l^{\prime },m}^{n,n^{\prime },\beta } \end{array}\right].$$

Equations 22 and 23 denote *N*_*x*_*N*_*l*_square matrices, where matrix (Equation 23) is upper triangular. In this manner, component matrices (Equations 24 and 25) are of the dimension *N*_*l*_×*N*_*l*_. Additionally, matrix $\varepsilon_{i^{\prime },j^{\prime }}$ always denotes diagonal matrix, while $h_{i^{\prime },j^{\prime }}$ matrix is much more complex, with possible nonzero elements at every position. Please note that some of the $h_{l,l^{\prime },m}^{n,n^{\prime },\beta }$ elements can vanish due to symmetry conditions and simplify the notation of the $h_{i^{\prime },j^{\prime }}$ matrix.

## Appendix 2

### Partitioning technique

The partitioning technique is a suitable method which allows to avoid the singularity problem of the ***H***_*N*,*N*−1_ and $H_{N,N-1}^{†}$ matrices and calculates only nontrivial solutions of Equation 10. Detailed discussion of the partitioning technique is presented in the work of Khomyakov and Brocks [71], and this section gives only our short remarks on this method.

Following studies from Khomyakov and Brocks [71], Equation 10 is partitioned into two parts of *D*_1_−*D*_2_ and *D*_2_ sizes where

(26) $$D_{1}=N_{x}N_{l},$$

and

(27) $$D_{2}=N_{n}N_{l}.$$

In Equation 27, parameter *N*_*n*_stands for the order of nearest-neighbor interactions assumed in calculations, e.g., *N*_*n*_=1 for the first nearest-neighbor interactions. On the basis of Equations 26 and 27, the reduced 2*N*_*l*_eigenvalue problem is written as follows:

(28) $$\begin{array}{lll} & \left[\left[\begin{array}{ll} A_{1,1} & A_{1,2}\\ I_{2,2} & 0 \end{array}\right]-z\left[\begin{array}{ll} B_{1,1} & B_{1,2}\\ 0 & I_{2,2} \end{array}\right]\right]\; & \;\\ & \times \left[\begin{array}{l} c_{2}(x_{N},k)\\ c_{2}(x_{N-1},k) \end{array}\right]=0.\; \end{array}$$

At this point, we correct the misprint from the study of Khomyakov and Brocks [71] and write the submatrices of Equation 28 in the following form:

(29) $$A_{1,1}=EI_{2,2}-E_{2,2}-E_{2,1}{\left[EI_{1,1}-E_{1,1}\right]}^{-1}E_{1,2},$$

(30) $$A_{1,2}=-H_{2,2}-E_{2,1}{\left[EI_{1,1}-E_{1,1}\right]}^{-1}H_{1,2},$$

(31) $$B_{1,1}=H_{2,2}^{†}+H_{1,2}^{†}{\left[EI_{1,1}-E_{1,1}\right]}^{-1}E_{1,2},$$

(32) $$B_{1,2}=H_{1,2}{\left[EI_{1,1}-E_{1,1}\right]}^{-1}H_{1,2}.$$

Please note that the reduced problem of Equation 28 gives 2*N*_*l*_ eigenvalues with 2*N*_*l*_ corresponding eigenvectors; this *N*_*x*_times less than can be expected from a physical point of view. Nevertheless, those solutions can be easily separated into *N*_*x*_*N*_*l*_ eigenvalues and *N*_*x*_*N*_*l*_eigenvectors of a purely physical character.

## Appendix 3

### Explicit forms of the **M**_i,j_, $M_{1}^{\mathrm{in}}$, and $M_{2}^{\mathrm{in}}$ components

The submatrices of the *matched*(*D* + 2)×(*D* + 2) square matrix ***M***in Equation 16, for a given *i* and *j* indices, are given as follows:

(33) $$M_{i,j}=EI-E_{i,i}\;\;\text{for}\;\;\left\{\begin{array}{l} i=j\\ D>i>1\\ D>j>1 \end{array}\right),$$

(34) $$M_{i,j}=-H_{i,i-1}\;\;\text{for}\;\;\left\{\begin{array}{l} i\neq j\\ i>2\\ j=i-1 \end{array}\right),$$

(35) $$M_{i,j}=-H_{i,i-1}^{†}\;\text{for}\;\left\{\begin{array}{l} i\neq j\\ i<D+1\\ j=i+1 \end{array}\right),$$

except for the submatrices which describe the boundary atoms of the system and those that are expressed in the following manner:

(36) $$\begin{array}{lll} & M_{1,1}=-H_{-1,-2}c_{l}(r_{n},z_{\gamma^{\prime }},E_{\gamma })z_{\gamma^{\prime }}^{2}\; & \;\\ & +\left(EI-E_{-1,-1}\right)c_{l}(r_{n},z_{\gamma^{\prime }},E_{\gamma })z_{\gamma^{\prime }},\; & \;\\ & M_{2,1}=-H_{0,-1}c_{l}(r_{n},z_{\gamma^{\prime }},E_{\gamma })z_{\gamma^{\prime }},\; & \;\\ & M_{D+1,D+2}=-H_{D-1,D}^{†}c_{l}(r_{n},z_{\gamma^{\prime }},E_{\gamma })z_{\gamma^{\prime }}^{D},\; & \;\\ & M_{D+2,D+2}=-H_{D,D+1}c_{l}(r_{n},z_{\gamma^{\prime }},E_{\gamma })z_{\gamma^{\prime }}^{D+1}\; & \;\\ & +\left(EI-E_{D,D}\right)c_{l}(r_{n},z_{\gamma^{\prime }},E_{\gamma })z_{\gamma^{\prime }}^{D}.\; \end{array}$$

Finally, the $M_{1}^{\mathrm{in}}$ and $M_{2}^{\mathrm{in}}$ of Equation 16 vector components are written as follows:

(37) $$\begin{array}{lll} M_{1}^{\mathrm{in}} & =H_{-1,-2}c_{l}(r_{n},z_{\gamma },E_{\gamma })z_{\gamma }^{-2}\; & \;\\ & \;+\left(EI-E_{-1,-1}\right)c_{l}(r_{n},z_{\gamma },E_{\gamma })z_{\gamma }^{-1},\; \end{array}$$

and

(38) $$M_{2}^{\mathrm{in}}=-H_{0,-1}c_{l}(r_{n},z_{\gamma },E_{\gamma })z^{-1}.$$

## Appendix 4

### Group velocities

As specified in the ‘Phase field matching theory’ subsection, the group velocities for individual states can be calculated on the basis of Equation 6 rewritten in the following manner:

(39) $$\left[vI-V\right]v(k,E)=0,$$

where *v* denotes the eigenvalues of Equation 39 which yields all required electron group velocities for each propagating state. Further, ***V***is the *N*_*x*_×*N*_*l*_size matrix of the following form:

(40) $$V=\frac{\partial M_{d}}{\partial k}.$$

Finally, ***v***(***R***_*N*_,***k***) stands for eigenvectors of the problem of Equation 39. We note that, usually, Equation 40 includes the constant part *d*_*β*_/h, where *h* is the Planck constant. However, for the purpose of electronic conductance calculations within the PFMT approach, this term can be omitted due to the fact that only the ratios of the given group velocities are important (please see Equations 17 and 18).

## Competing interests

The authors declare that they have no competing interests.

## Authors, contributions

DS participated in the design of this study, in analytical calculations, and in writing the code for numerical calculations, carried out numerical calculations, drafted the manuscript, and participated in writing the final version of the manuscript. AK coordinated this study, participated in its design and analytical calculations, and in writing the final version of the manuscript. ZB participated in the design of this study, its coordination, and substantial critical revision of the final version of the manuscript. RS participated in writing the code for numerical calculations and substantial critical revision of the final version of the manuscript. MAG participated in the substantial critical revision of the final version of the manuscript. All authors read and approved the final manuscript.
